# Induction of tumor inhibitory anti-angiogenic response through immunization with interferon Gamma primed placental endothelial cells: ValloVax™

**DOI:** 10.1186/s12967-015-0441-0

**Published:** 2015-03-14

**Authors:** Thomas E Ichim, Shuang Li, Hong Ma, Yuliya V Yurova, Julia S Szymanski, Amit N Patel, Santosh Kesari, Wei-Ping Min, Samuel C Wagner

**Affiliations:** Batu Biologics Inc, San Diego, 9255 Towne Centre Drive, Suite 450, San Diego, CA 92121 USA; Department of Endocrinology, The Affiliated Zhongshan Hospital of Dalian University, Dalian, 116001 China; Nova Southeastern University, Fort Lauderdale, Florida USA; Department of Surgery, University of Utah, Salt Lake City, Utah; Department of Neurosciences, University of California San Diego, 9500 Gilman Dr., MSC 0752, La Jolla, San Diego, CA 92093-0752 USA; Translational Neuro-Oncology Laboratories, Moores Cancer Center, University of California San Diego, 3855 Health Sciences Dr., MSC 0819, La Jolla, San Diego, CA 92093-0819 USA; Department of Immunology, University of Western Ontario, London, Ontario Canada

## Abstract

**Background:**

While the concept of angiogenesis blockade as a therapeutic intervention for cancer has been repeatedly demonstrated, the full promise of this approach has yet to be realized. Specifically, drugs such as VEGF-blocking antibodies or kinase inhibitors suffer from the drawbacks of resistance development, as well as off-target toxicities. Previous studies have demonstrated feasibility of specifically inducing immunity towards tumor endothelium without consequences of systemic autoimmunity in both animal models and clinical settings.

**Method:**

Placenta-derived endothelial cells were isolated and pretreated with interferon gamma to enhance immunogenicity. Syngeneic mice received subcutaneous administration of B16 melanoma, 4 T1 mammary carcinoma, and Lewis Lung Carcinoma (LLC), followed by administration of control saline, control placental endothelial cells, and interferon gamma primed endothelial cells (ValloVax™). Tumor volume was quantified. An LLC metastasis model was also established and treated under similar conditions. Furthermore, a safety analysis in non-tumor bearing mice bracketing the proposed clinical dose was conducted.

**Results:**

ValloVax™ immunization led to significant reduction of tumor growth and metastasis as compared to administration of non-treated placental endothelial cells. Mitotic inactivation by formalin fixation or irradiation preserved tumor inhibitory activity. Twenty-eight day evaluation of healthy male and female mice immunized with ValloVax™ resulted in no abnormalities or organ toxicities.

**Conclusion:**

Given the established rationale behind the potential therapeutic benefit of inhibiting tumor angiogenesis as a treatment for cancer, immunization against a variety of endothelial cell antigens may produce the best clinical response, enhancing efficacy and reducing the likelihood of the development of treatment resistance. These data support the clinical evaluation of irradiated ValloVax™ as an anti-angiogenic cancer vaccine.

**Electronic supplementary material:**

The online version of this article (doi:10.1186/s12967-015-0441-0) contains supplementary material, which is available to authorized users.

## Background

Tumors utilize a variety of molecular mechanisms to evade the immune response, including loss of tumor specific antigens [1-3], suppression of antigen presenting machinery such as transporter associated protein and MHC expression [4-7], and the production of immunosuppressive factors, both soluble and surface bound [8]. Additionally, tumors lack expression of co-stimulatory molecules critical for the activation of naïve T cells, and suppress the expression of these molecules on antigen presenting cells [9]. Tolerogenic means elaborated by the tumor inhibit T cell activation while creating a microenvironment conducive to T cell exhaustion. Poor T cell function in the tumor microenvironment allows tumors to escape immune-mediated destruction promoting the developent of treatment resistance through immunoediting [10]. The ability of tumors to escape immune pressure and sculpt their immunogenic phenotype to evade immune destruction makes it exceedingly difficult to develop effective immunotherapies targeting tumor-derived antigens. A novel approach towards inducing anti-tumor immunity would be to target not the tumor itself, but the blood supply feeding the tumor, an essential mechanism of tumor growth.

Immunological targeting of tumor endothelium is appealing based on: a) For every tumor endothelial cell therapeutically neutralized approximately 200–300 tumor cells perish, thus reducing ability of tumors to lose expression of antigens; b) The immune system is in direct contact with the tumor endothelium, while immune access inside tumors is difficult due to areas of necrosis and high interstitial pressure; and c) Demonstrated prior efficacy of other anti-angiogenesis inhibitory compounds such as bevacizumab [11,12]. Furthermore, the elevated expression of Fas Ligand on the tumor endothelium mediates the selective killing of CD8+ Tumor Infiltrating Lymphocytes (TIL) allowing for a predominance of FoxP3+ T regulatory cells (Treg) to infiltrate the tumor microenvironment, demonstrating that the tumor blood vessels act as an immunological barrier promoting tumor tolerance [13]. Immune-mediated destruction of the tumor endothelium has been shown to significantly increase TILs in mouse models, which was correlated with tumor regression [14]. Another further potential benefit of targeting the tumor associated vasculature is the potential of sensitizing tumors to radiotherapy [15], in part due to the selective thrombotic and apoptotic effects irradiation has on the tumor vasculature [16-19]. Current tyrosine kinase inhibitors blocking angiogenesis systemically inhibit pro-angiogenic factors such as Vascular Endothelial Growth Factor (VEGF) or Angiopoetin, slowing blood vessel formation without differentiating between tumor and healthy angiogenesis. However, therapeutics that stimulate direct damage to the tumor endothelium have been shown to activate the coagulation cascade, effectively cutting off blood supply to the tumor and creating a hypoxic microenvironment conducive to necrosis and tumor regression [20]. A more effective anti-angiogenesis approach may be to stimulate selective killing of the tumor endothelium through immunotherapeutic vaccines.

A fundamental question determining feasibility of vaccine-induced killing of tumor vasculature is whether antigens exist on the tumor endothelium that are not expressed on physiologically normal blood vessels, and whether immunity could be raised against such antigens. A few tumor endothelium-specific antigens have been reported. The roundabout receptor (ROBO)-4 is a transmembrane protein that was originally found to orchestrate the neuronal guidance mechanism of the nervous system [21]. ROBO4 was found to be selectively expressed on tumor endothelial cells but not healthy vasculature [22]. Zhuang et al. demonstrated that mice immunized with the extracellular domain of mouse Robo4, showed a strong antibody response to Robo4, with no objectively detectable adverse effects on health, including normal menstruation and wound healing. Robo4 vaccinated mice showed impaired fibrovascular invasion and angiogenesis in a rodent sponge implantation assay, as well as a reduced growth of implanted syngeneic Lewis lung carcinoma. The anti-tumor effect of Robo4 vaccination was present in CD8 deficient mice but absent in B cell or IgG1 knockout mice, suggesting antibody-dependent cell mediated cytotoxicity as the anti-vascular/anti-tumor mechanism [23]. Another antigen that is more ubiquitously found throughout the body, but with higher expression on tumor endothelial cells is the VEGF receptor 2 (VEGFR2) which is typically found on hematopoietic stem cells and endothelial progenitor cells [24-29]. Despite expression on non-malignant tissue, successful induction of antitumor immunity has been demonstrated using various immunization means against this antigen. Yan et al. utilized irradiated AdVEGFR2-infected cell vaccine-based immunotherapy in the weakly immunogenic and highly metastatic 4 T1 murine mammary cancer model. Lethally irradiated, virus-infected 4 T1 cells were used as vaccines. Vaccination with lethally irradiated AdVEGFR2-infected 4 T1 cells inhibited subsequent tumor growth and pulmonary metastasis compared with challenge inoculations. Angiogenesis was inhibited, and the number of CD8+ T lymphocytes was increased within the tumors. Antitumor activity was also caused by the adoptive transfer of isolated spleen lymphocytes, thus demonstrating induction of tumor specific immunity [30]. Other approaches have been utilized to induce immunity to VEGFR2, which resulted in induction of tumor regression without systemic toxicities [31-36]. Tumor endothelial marker 1 or endosialin is another antigen found selectively on the tumor vasculature. Facciponte et al. demonstrated that a DNA vaccination targeting endosialin reduced tumor vascularity, increased CD3+ T cell infiltration, and was correlated with significant inhibition of tumor growth. Epitope spreading to tumor antigens following the initial immune response against the tumor vasculature gives evidence that targeting the tumor endothelium may activate a cascade of pathways conducive to tumor regression. Additionally, the DNA vaccination against endosialin did not affect other angiogenesis dependent physiological processes, exhibiting no adverse effects on menstruation, embryonic development, pregnancy, and wound healing in mouse models [14]. Other markers associated with tumor blood vessels have been utilized therapeutically in animal models for vaccination purposes including survivin [37-39], xenogeneic FGF2R [40], VEGF [41], VEGF-R2 [42], MMP-2 [43], and endoglin [44,45].

Although tumor endothelial cells are more genetically stable then the tumor cells, thus reducing the possibility of immune mediated antigen loss, some mutational activity has been reported in tumor associated vascular cells [46,47]. Accordingly, a polyvalent vaccine approach targeting the immune system toward a plethora of endothelial cell antigens specific to the tumor endothelium may be more effective. With this approach comes a heightened theoretical risk of autoimmunity. Despite these theoretical concerns successful immunization against tumor endothelium has been performed utilizing Human Umbilical Vein Endothelial Cells (HUVEC). Wei et al. demonstrated that vaccination of mice with fixed xenogeneic whole endothelial cells (in the form of HUVEC) as a vaccine was effective in affording protection from tumor growth, inducing regression of established tumors, and prolonging the survival of tumor-bearing mice. Additionally, the authors found that, immunity targeted to tumor vasculature was induced and was responsible for the anti-tumor activity, which was not associated with any noticeable toxicity toward non-malignant tissues [48,49]. From a clinical perspective, a 17 patient trial demonstrated that HUVEC vaccine therapy significantly prolonged tumor doubling time and inhibited tumor growth in patients with recurrent glioblastoma, inducing both cellular and humoral responses against the tumor vasculature without any adverse events or noticeable toxicities [50]. The clinical efficacy of using HUVEC vaccination to break tolerance to tumor angiogenesis has also been demonstrated in patients with colorectal cancer and malignant brain tumors without any observable adverse effects on healthy angiogenesis [20].

In this current study it was demonstrated that placental endothelial cells that are interferon gamma primed potently inhibit tumor growth in 3 histologically distinct animal models, as well as suppress pulmonary metastasis subsequent to intravenous tumor administration. Furthermore, the therapeutic effect was retained when placental endothelial cells have been mitotically inactivated by either formalin or irradiation. The success of this new approach may provide a new way to develop clinical effective placental cells vaccination against a wide variety of tumors by targeting tumor angiogenesis.

## Materials and methods

### Animals and cells

Female C57BL/6 and BALB/c mice aged 8–12 weeks were purchased from The Jackson Laboratory. Animals were housed under conventional conditions at the Animal Care Facility, University of Western Ontario, and were cared for in accordance with the guidelines established by the Canadian Council on Animal Care. A murine melanoma cell line established from a C57BL/6 mouse and designated B16F10 was obtained from the American Type Culture Collection (ATCC) and was maintained in RPMI 1640 medium (Sigma-Aldrich) with 10% FBS, l-glutamine, penicillin, and streptomycin at 37°C in 5% CO_2_. The murine mammary carcinoma 4 T1 cells (ATCC) were grown DMEM medium (Sigma-Aldrich) with 10% FBS, l-glutamine, penicillin, and streptomycin at 37°C in 5% CO_2_. Lewis Lung Carcinoma (LLC) is a murine lung carcinoma originating from C57/BL6 mice. The cells were maintained in RPMI 1640 supplemented with 10% fetal bovine serum, 2 mM glutamine (Gibco-BRL, Life Technologies, Inc.). The cell line was cultured at 37°C in a 5% incubator.

### Preparation of vaccine

Full term human placentas were collected from delivery room under informed consent. Fetal membranes were manually peeled back and the villous tissue is isolated from the placental structure. Villous tissue was subsequently washed with cold saline to remove blood and scissors used to mechanically digest the tissue. Lots of 25 grams of minced tissue were incubated with approximately 50 ml of HBSS with 25 mM of HEPES and 0.28% collagenase, 0.25% dispase, and 0.01% DNAse at 37 Celsius. The mixture of minced placental villus tissue and digesting solution was incubated under stirring conditions for three incubation periods of 20 minutes each. Ten minutes after the first incubation period and immediately after the second and third incubation periods, the DNAse was added to make up a total concentration of DNase, by volume, of 0.01%. In the first and second incubations, the incubation flask is set at an angle, and the tissue fragments allowed to settle for approximately 1 minute, with 35 ml of the supernatant cell suspension being collected and replaced by 38 ml (after the first digestion) or 28 ml (after the second digestion) of fresh digestion solution. After the third digestion the whole supernatant was collected. The supernatant collected from all three incubations was then pooled and is poured through approximately four layers of sterile gauze and through one layer of 70 micrometer polyester mesh. The filtered solution was then centrifuged for 1000 g for 10 minutes through diluted new born calf serum, said new born calf serum diluted at a ratio of 1 volume saline to 7 volumes of new born calf serum. The pooled pellet was then resuspended in 35 ml of warm DMEM with 25 mM HEPES containing 5 mg DNase I. The suspension was subsequently mixed with 10 ml of 90% Percoll to give a final density of 1.027 g/ml and centrifuged at 550 g for 10 minutes with the centrifuge brake off. The pellet was then washed in HBSS and cells incubated for 48 hours in complete DMEM media. After 3–4 passages cells were incubating in media containing 100 IU of IFN-gamma per ml. Subsequent to incubation cells were either used: a) unmanipulated; b) used as a lysate, with 10 freeze thaw cycles in liquid nitrogen, subsequent to which lysate was filtered through a 0.2 micron filter; c) mitotically inactivated by irradiation at 10 Gy; or d) inactivated by fixation in 0.5% formalin and subsequently washed.

### Immunization schedules and tumor assessment

For induction of tumor growth, 5 × 10^5^ B16, LLC, or 4 T1 cells, American Type Culture Collection (Manassas, VA) cells were injected subcutaneously into the hind limb flank. Four weekly vaccinations of 5 × 10^5^ test cells were administered subcutaneously on the contralateral side to which tumors were administered. Vaccination was performed on the day of tumor inoculation and on days 7, 14, and 21. Tumor growth was assessed every 3 days by two measurements of perpendicular diameters by a caliper, and animals were sacrificed when tumors reached a size of 1 cm in any direction. Tumor volume was calculated by the following formula: (the shortest diameter^2^ × the longest diameter)/2.

## Results

### Successful utilization of placental endothelial cells in induction of anticancer immunity regardless of tumor type

**Figure 1:**
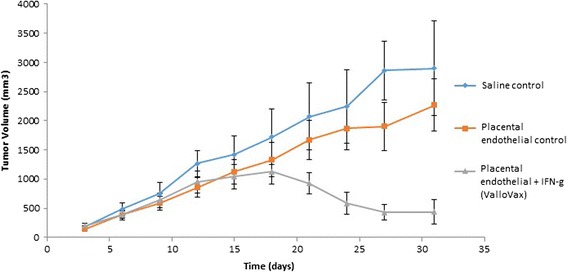
**ValloVax™ treatment inhibits B16 melanoma growth.** Female C57BL/6 mice (10 mice per group) were immunized with saline (diamond), or 5 × 10(5) placental endothelium cells (square) or placental endothelium cells pretreated with interferon gamma (triangle) on days 0, 7, 14, and 21. Tumor growth was initiated by subcutaneous administration of 5 × 10(5) B16 cells and quantified every third day.

**Figure 2:**
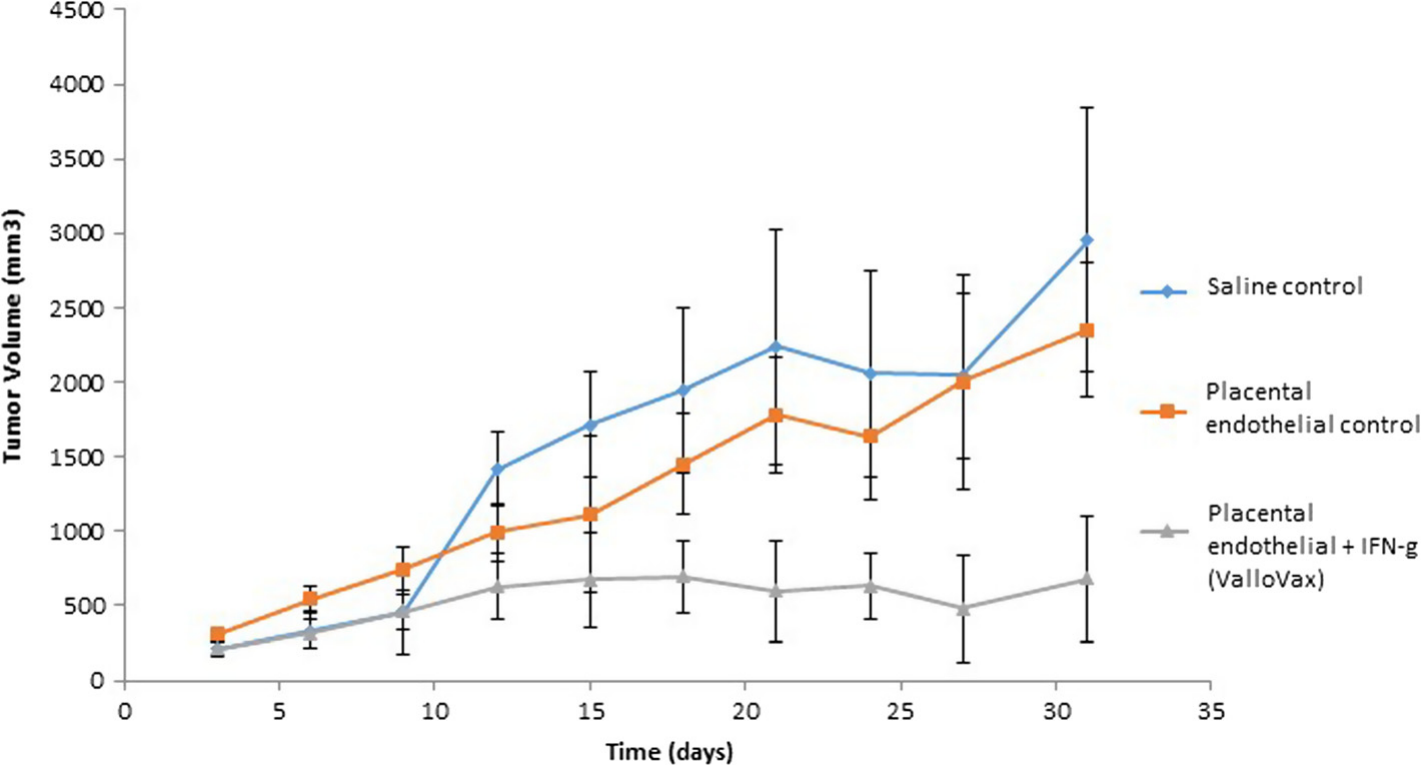
**ValloVax™ treatment inhibits 4 T1 mammary carcinoma growth.** Female BALB/c mice (10 mice per group) were immunized with saline (diamond), or 5 × 10(5) placental endothelium cells (square) or placental endothelium cells pretreated with interferon gamma (triangle) on days 0, 7, 14, and 21. Tumor growth was initiated by subcutaneous administration of 5 × 10(5) 4 T1 cells and quantified every third day.

**Figure 3:**
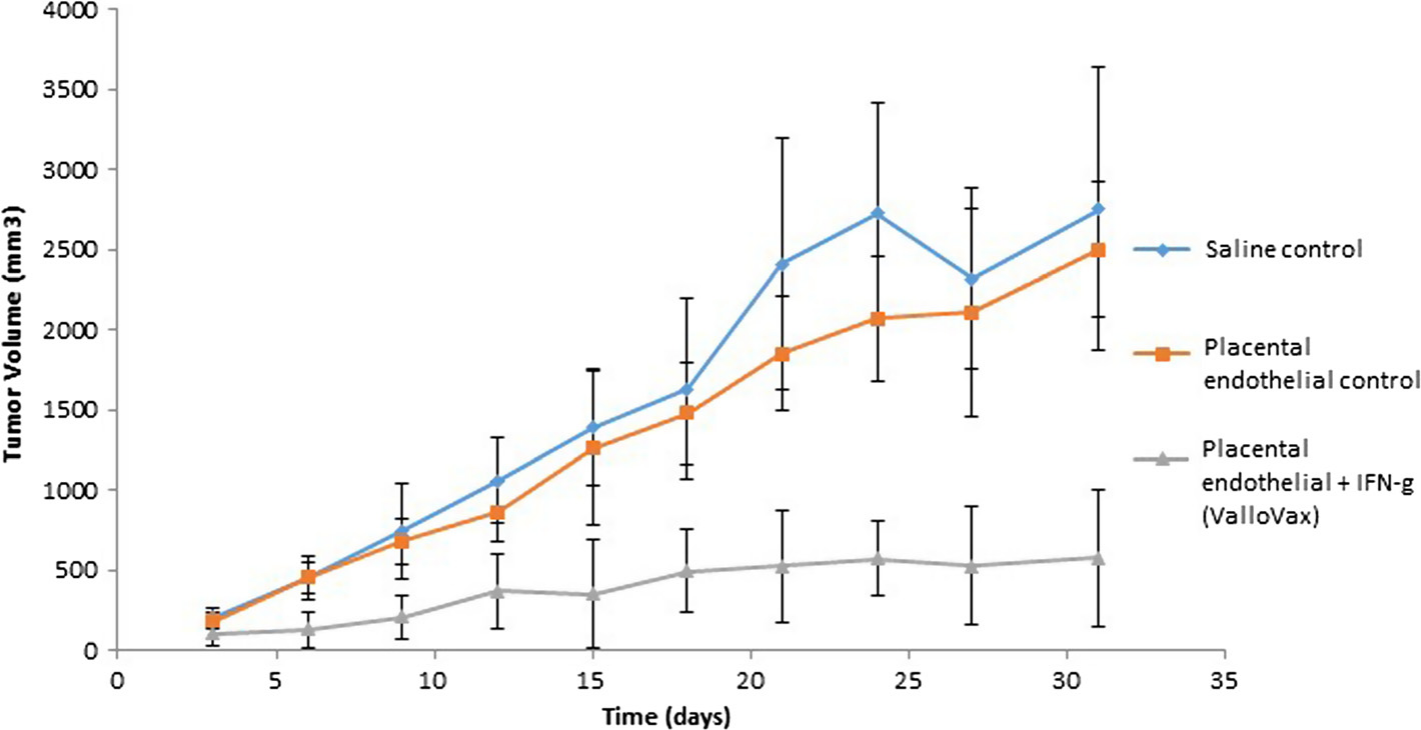
**ValloVax™ treatment inhibits LCC lung carcinoma growth.** Female C57BL/6 mice (10 mice per group) were immunized with saline (diamond), or 5 × 10(5) placental endothelium cells (square) or placental endothelium cells pretreated with interferon gamma (triangle) on days 0, 7, 14, and 21. Tumor growth was initiated by subcutaneous administration of 5 × 10(5) LLC cells and quantified every third day.

While it has previously been demonstrated that vaccination with autologous and allogeneic endothelial cells results in tumor regression [51-55], and safety of this approach has been reported in clinical studies [20], current means of extracting endothelial cells are limited to the need for tissue culture expansion. Generally endothelial cells proliferate poorly *in vitro* and require the addition of recombinant growth factors that add expense, as well as possibility of contamination during production of clinical grade production. Accordingly, a more practical source of endothelium would be the placental body, which contains up to 2–10 billion primary endothelial cells per placenta [56-58]. We immunized mice bearing LLC, B16 and 4 T1 cells. The immunization schedule was a therapeutic one in that time of immunization occurred concurrently with the administration of tumor challenge. As seen in Figures 1, 2 and 3, a trend towards reduction of tumor growth was observed with non-IFN-gamma pretreated endothelial cells, while a potent reduction of tumor growth was seen in animals treated with cells that were first stimulated with interferon gamma. Interferon gamma pretreatment was shown to upregulate HLA I and HLA II (data not shown).

### Mitotically inactivated endothelial cell vaccine retains antitumor activity across histologically different tumors

**Figure 4:**
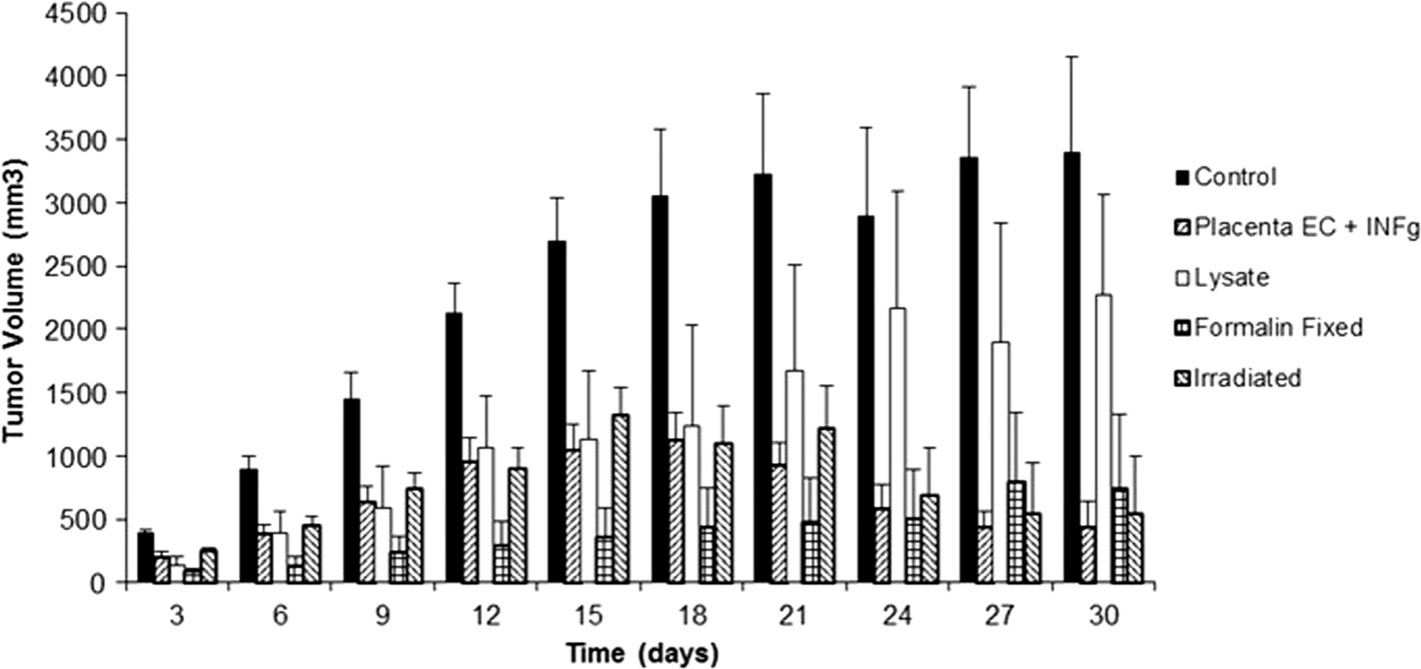
**Mitotically inactivated ValloVax™ retains activity against B16 melanoma growth.** Female C57BL/6 mice (10 mice per group) were immunized with saline, ValloVax™, ValloVax™ lysate, formalin fixed ValloVax™, or irradiated Vallovax™ at a concentration of 5 × 10(5) cells or cell equivalents on days 0, 7, 14, and 21. Tumor growth was initiated by subcutaneous administration of 5 × 10(5) B16 cells and quantified every third day.

**Figure 5:**
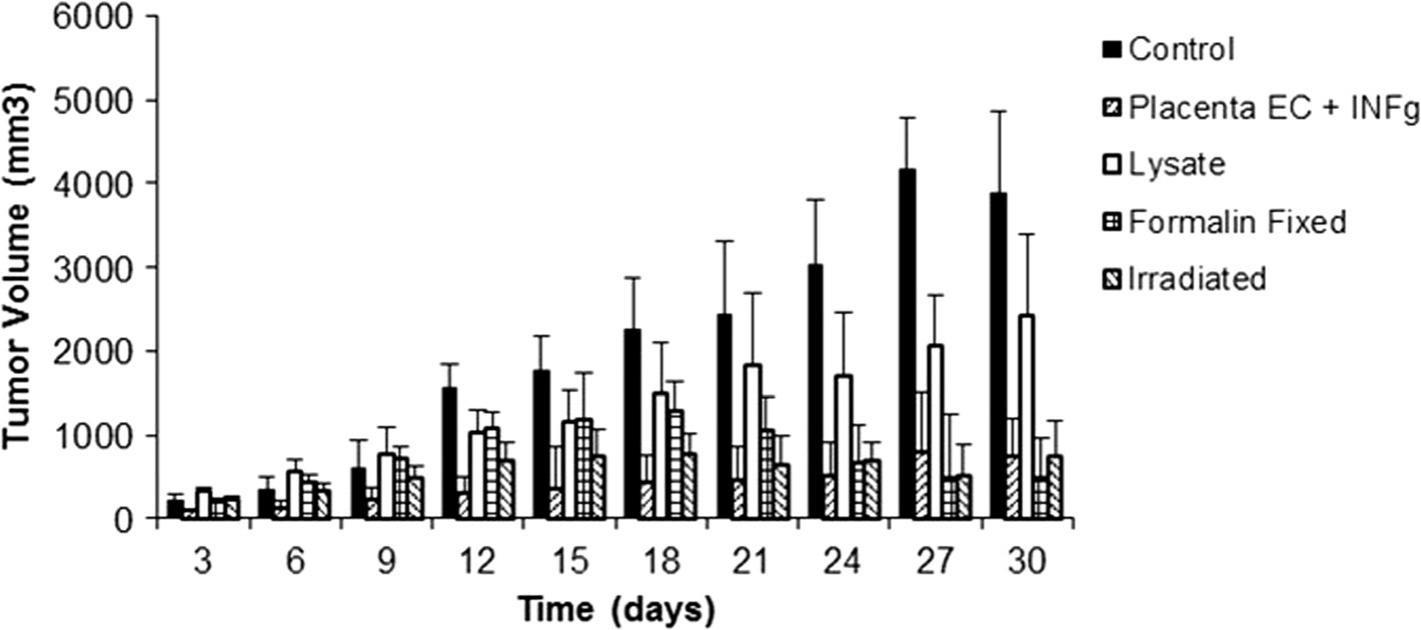
**Mitotically inactivated ValloVax™ retains activity against 4 T1 mammary carcinoma growth.** Female BALB/c mice (10 mice per group) were immunized with saline, ValloVax™, ValloVax™ lysate, formalin fixed ValloVax™, or irradiated Vallovax™ at a concentration of 5 × 10(5) cells or cell equivalents on days 0, 7, 14, and 21. Tumor growth was initiated by subcutaneous administration of 5 × 10(5) 4 T1 cells and quantified every third day.

**Figure 6:**
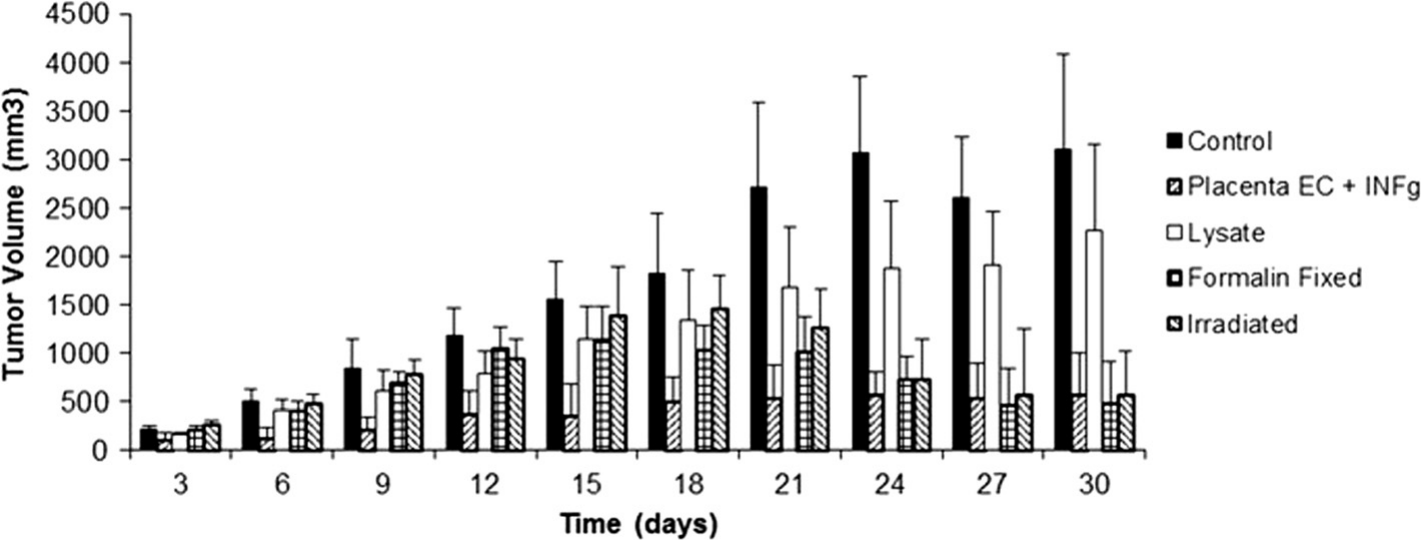
**Mitotically inactivated ValloVax™ Retains™ activity against LLC lung carcinoma.** Female C57BL/6 mice (10 mice per group) were immunized with saline, ValloVax™, ValloVax™ lysate, formalin fixed ValloVax™, or irradiated Vallovax™ at a concentration of 5 × 10(5) cells or cell equivalents on days 0, 7, 14, and 21. Tumor growth was initiated by subcutaneous administration of 5 × 10(5) LLC cells and quantified every third day.

For clinical development of a cancer angiogenesis vaccine, it is imperative to generate cells that are mitotically inactivated. Part of the reason for this is that administration of viable endothelial cells could potentially result in acceleration of tumor growth through enhancement of angiogenesis [59]. Furthermore, in previous clinical trials, endothelial cells were pretreated with a fixative to avoid this potential issue [20]. As seen in Figures 4, 5 and 6, for all tumor models tested, mitotic inactivation utilizing irradiation was mildly superior to formalin fixation. Additionally, no therapeutic effect was observed by administration of endothelial cell lysate, with activity being retained in activated endothelial cells.

### Endothelial cell vaccine inhibit tumor metastasis

**Figure 7:**
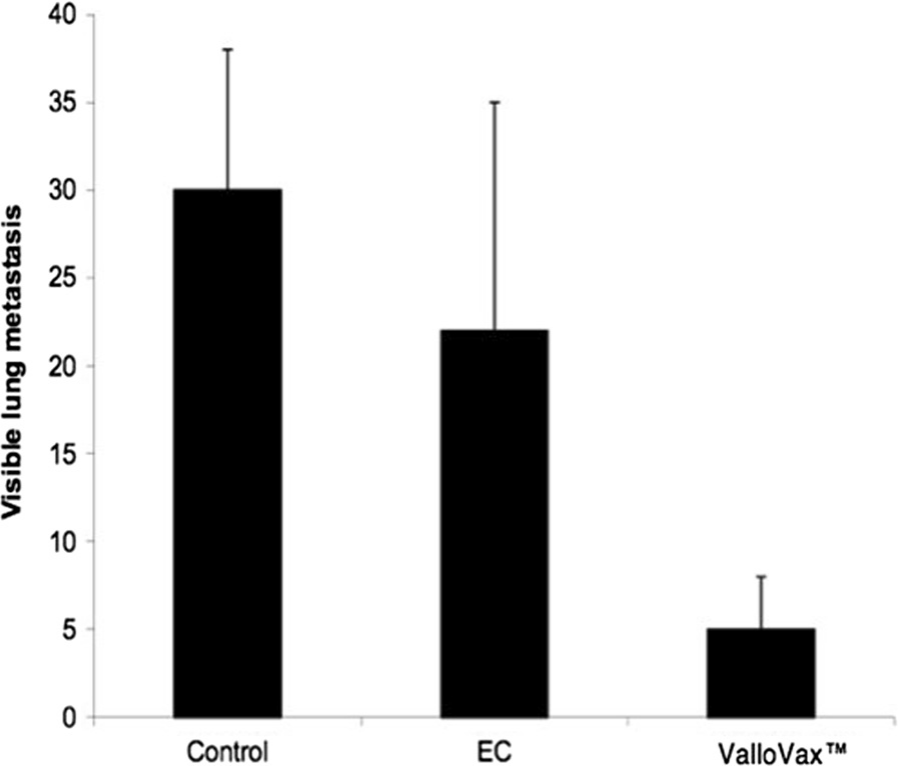
**Reduction in lung metastasis after ValloVax™ immunization.** Female C57BL/6 mice (10 mice per group) were immunized with saline, placental endothelial cells, and placental endothelial cells pretreated with IFN-gamma. Immunization was performed subcutaneously at same time as 5 × 10(5) LLC cells were administered intravenously. Mice were sacrificed after 3 weeks and lung colonies were quantified by counting per visual field.

In addition to reduction of tumor size growth, an important aspect of cancer immunization is reduction in lung metastasis. Importantly, in the utilization of vaccines against cancer, the ability of the immune system to seek and destroy metastatic cells is one of the key enticing factors of this approach. When intravenous administration of LLC was performed in C57BL/6 mice, immunization with ValloVax™ resulted in inhibition of tumor lung metastasis colonies (Figure 7). The inhibition was significantly more profound when placental endothelial cells were treated with interferon gamma.

### Safety evaluation

Seven male and female mice per group were treated with control, or 500,000, 2 million or 4 million ValloVax™ irradiated cells subcutaneously. Cells were administrated as in the therapeutic protocol, in that they were given on day 0, 7, 14, and 21. Body weights were calculated every three days and organ sizes, biochemical and hematological parameters were evaluated. No significant deviation was noted, nor were signs of autoimmunity present (online Additional file 1). These preclinical data support the safety of the irradiated ValloVax™ approach.

## Discussion

The concept of targeting tumor associated endothelium has been a holy grail of cancer therapists since the original work of Judah Folkman demonstrated that tumors cannot grow more than 1–2 millimeters without the stimulation of new blood vessel formation (angiogenesis) [60]. Specifically blocking angiogenesis is intellectually enticing because the tumor-associated endothelium is derived from non-mutated tissue; therefore the possibility of development of a drug resistant phenotype is very low. Unfortunately several angiogenesis-targeting drugs that have demonstrated promising results in animal trials have failed in pivotal clinical trials. Examples include angiostatin, endostatin, and shark cartilage extract (Neovastat) [61,62]. More recent studies using the VEGF pathway blocking antibody, bevacizumab (Avastin), have demonstrated positive results in specific types of tumors, which led to regulatory approvals [63]. Unfortunately the use of these antibodies requires co-administration of chemotherapy, and is associated with signification toxicity [64]. Furthermore, for reasons unknown, bevacizumab is ineffective in several tumor types, and in the tumors that it is effective, resistance often ensues, limiting long-term therapeutic utility [65].

The possibility of inducing selective immunity to proliferating blood vessels has been previously reported in animal models as well as pilot clinical trials. Unfortunately, a major limiting factor to clinical implementation has been the utilization of HUVEC cells as an antigenic source, which is limited in availability. Here we utilized placental derived endothelial cells as a source of antigen found on proliferating endothelium such as the tumor. The utilization of placenta derived tissues for immunization to cancer was initially introduced in the 1970s by Dr. Valentin Govallo (reviewed in Harandi [66]) who demonstrated that immunity to placental trophoblast extract resulted in reduction of immune suppression using the PHA stimulation assay, as well as radiological tumor reductions. Dr. Govallo noted the immunological similarities between pregnancy and cancer. Later along with the advancement in molecular biology development founding placental cells and tumor endothelial cells share the molecules of angiogenesis such as VEGF, placental growth factor, angiopoietin, FGF, EGF, and TGF-beta, and as well placental endothelium expresses many of the novel tumor endothelial markers (TEM) such as ROBO4 [67], CLEC14A [68], and endosialin [69] suggested parallels between the placental and tumor microenvironment, namely immune suppression, active angiogenesis, and the secretion of matrix metalloproteinase associated with metastasis, not just a functional, but also a molecular homology between placenta, tumor cells and tumor-associated endothelium. Based on the safety and possible efficacy of the Govallo vaccine, we sought to utilize placental endothelial cells as a polyvalent antigenic source for stimulation of immunity against proliferating endothelial cells that have been primed with interferon gamma to stimulate immunogenicity. We termed this product “ValloVax™”.

Here we demonstrate the therapeutic activity of ValloVax™ against a wide range of histologically distinct tumor types, suggesting that the effect is acting against new blood vessels and not against shared tumor antigens. Additionally, the main concern of utilization of an antiendothelial vaccine would be the possibility of inducing autoimmunity against the endothelium. This has not been observed in the 28 day safety study. We have demonstrated that tumor inhibiting activity was preserved when cells were mitotically inactivated, however was substantially reduced when cell lysate was utilized. It is important to note that adjuvants were not administered as part of the vaccination inoculum. Accordingly, manipulation of the vaccine administration either by modification of dosage or frequency may induce more potent therapeutic responses. One of the deficiencies of the current study is the lack of direct demonstration that inhibition of endothelial proliferation was responsible for reduction in tumors. Although it is unlikely that direct tumor immunity was induced to all three tumor types assessed by the administration of ValloVax™, this possibility cannot be excluded. Supporting the possibility that anti-endothelial immunity was induced is data demonstrating sera of immunized mice was able to inhibit proliferation of endothelial cells in vitro. These data are currently the subject of an additional manuscript looking at more detailed mechanisms of immunity and tumor biology. As well further studies on combination of ValloVax™ with standard of care treatments will be studied.

## Conclusion

Based on existing preclinical and clinical data demonstrating safety of endothelial cell vaccination, combined with the recent data described herein, ValloVax™ appears to be a promising antiangiogenic vaccine platform. Demonstration of efficacy in 3 different animal models supports possibility utilization against a broad spectrum of tumors.

## Additional file

Additional file 1:
**Seven male and female mice per group were treated with control, or 500,000, 2 million or 4 million ValloVax™ irradiated cells subcutaneously.** Cells were administrated on days 0, 7, 14, and 21. Body weight was assessed on days 1, 14 and 28, whereas, biochemical and hematological parameters were evaluated at the termination of the experiment on day 28.
