# Supplementary material for: Induction of tumor inhibitory anti-angiogenic response through immunization with interferon Gamma primed placental endothelial cells: ValloVax™
Source: J Transl Med. 2015 Mar 14;13:90. doi: 10.1186/s12967-015-0441-0 (PMC4363400; doi:10.1186/s12967-015-0441-0)
Supplement: Additional file 1: — Seven male and female mice per group were treated with control, or 500,000, 2 million or 4 million ValloVax™ irradiated cells subcutaneously. Cells were administrated on days 0, 7, 14, and 21. Body weight was assessed on days 1, 14 and 28, whereas, biochemical and hematological parameters were evaluated at the termination of the experiment on day 28. [file 12967_2015_441_MOESM1_ESM.docx]

**Additional file 1**

**Online Supplement**

**Experimental Design:** Seven healthy male and seven healthy female BALB/c mice of 6-8 weeks of age were administered ValloVax irradiated cells on day 0, day 7, day 14, and day 21 via the subcutaneous route. Animals were observed daily for signs of behavior modification, and were sacrificed at day 28 and safety parameters were evaluated. Group 1 = Saline; Group 2 = 500,000 ValloVax cells; Group 3 = 2 million ValloVax cells; Group 4 = 4 million ValloVax cells.

Assessments were made of weight changes on day 1, day 14, and day 28. Evaluation of hematological parameters, serum biochemistry parameters, and organ weights (heart, lungs, liver/gall bladder, kidneys, spleen, brain, ovaries/uterus) was performed at day 28 when animals were sacrificed.

**Outcome:** No significant changes were observed in animal weight, hematological parameters, or serum biochemistry parameters between treatment groups.

**TABLE 1. Summary of Female Body Weights (g) and Body Weight Change (g)**

**Mean + Standard Deviation (SD)**

| Group | Day 1 | |  | Day 14 | |  | Day 28 | |  |
| --- | --- | --- | --- | --- | --- | --- | --- | --- | --- |
|  | Mean | SD | N | Mean | SD | N | Mean | SD | N |
| 1 - Control | 20.2 | 0.6 | 8 | 22.0 | 1.0 | 8 | 23.7 | 0.4 | 8 |
| 2 | 20.7 | 0.9 | 8 | 22.3 | 0.8 | 8 | 23.7 | 0.9 | 8 |
| 3 | 20.0 | 0.7 | 8 | 20.8 | 0.4 | 7 | 22.3 | 0.7 | 8 |
| 4 | 20.0 | 0.6 | 8 | 20.7 | 0.6 | 8 | 21.9 | 0.5 | 8 |

**TABLE 2. Summary of Male Body Weights (g) and Body Weight Change (g)**

**Mean + Standard Deviation (SD)**

| Group | Day 1 | |  | Day 14 | |  | Day 28 | |  |
| --- | --- | --- | --- | --- | --- | --- | --- | --- | --- |
|  | Mean | SD | N | Mean | SD | N | Mean | SD | N |
| 1 - Control | 21.6 | 0.6 | 8 | 22.2 | 0.7 | 8 | 23.6 | 0.8 | 8 |
| 2 | 21.6 | 0.6 | 8 | 22.6 | 0.8 | 8 | 23.8 | 0.6 | 8 |
| 3 | 21.5 | 0.5 | 8 | 22.7 | 1.0 | 8 | 23.8 | 0.7 | 8 |
| 4 | 21.8 | 1.0 | 8 | 22.9 | 0.9 | 8 | 23.8 | 0.7 | 8 |

**TABLE 3. Summary of Day 28 Female Clinical Chemistry Values**

**Mean + Standard Deviation (SD)**

| Group | 1 – Control | | 2 | | 3 | | 4 | |
| --- | --- | --- | --- | --- | --- | --- | --- | --- |
| Mice/group | N = 7 | | N = 7 | | N = 7 | | N = 7 | |
|  | Mean | SD | Mean | SD | Mean | SD | Mean | SD |
| ALB (g/dL) | 3.5 | 1.4 | 3.0 | 0.7 | 3.0 | 0.3 | 3.9 | 0.4 |
| AST (IU/L) | 245.4 | 91.4 | 208.7 | 20.9 | 212.8 | 13.7 | 235.5 | 12.9 |
| ALT (IU/L) | 48.4 | 7.5 | 52.4 | 3.0 | 50.8 | 2.4 | 51.9 | 5.5 |
| TBIL (mg/dL) | 0.2 | 0.1 | 0.2 | 0.0 | 0.1 | 0.0 | 0.2 | 0.0 |
| BUN (mg/dL) | 24.4 | 3.3 | 26.9 | 3.4 | 22.7 | 1.7 | 26.5 | 2.5 |
| CREAT (mg/dL) | 0.2 | 0.0 | 0.1 | 0.0 | 0.2 | 0.0 | 0.2 | 0.0 |
| PHOS (mg/dL) | 9.3 | 1.1 | 9.5 | 0.9 | 10.6 | 1.2 | 9.6 | 1.5 |
| GLUC (mg/dL) | 101.8 | 6.5 | 98.7 | 3.3 | 100.2 | 6.8 | 99.1 | 2.6 |
| Ca^2+^ (mg/dL) | 9.4 | 1.3 | 9.6 | 0.9 | 9.4 | 1.2 | 9.9 | 1.0 |
| Mg^2+^ (mEq/L) | 1.5 | 0.4 | 1.4 | 0.2 | 1.5 | 0.4 | 1.3 | 0.2 |
| Na^+^ (mEq/L) | 157.6 | 26.5 | 148 | 4.5 | 162.1 | 17.3 | 139.3 | 6.7 |
| K^+^ (mEq/L) | 9.6 | 1.1 | 9.2 | 0.5 | 10.3 | 0.8 | 9.3 | 1.0 |
| Cl^-^ (mEq/L) | 110.3 | 12.2 | 100.8 | 8.6 | 117.1 | 19.0 | 121.8 | 6.2 |
| AMYLASE (IU/L) | 759.6 | 136.8 | 709.1 | 13.9 | 785.7 | 40.4 | 783.9 | 32.2 |
| LIPASE (IU/L) | 61.0 | 8.9 | 62.5 | 2.7 | 68.3 | 2.8 | 64.2 | 3.9 |
| CPK (IU/L) | 523.5 | 73.9 | 501.1 | 10.0 | 532.0 | 25.9 | 493.7 | 8.1 |

**TABLE 4. Summary of Day 28 Male Clinical Chemistry Values**

**Mean + Standard Deviation (SD)**

| Group | 1 - Control | | 2 | | 3 | | 4 | |
| --- | --- | --- | --- | --- | --- | --- | --- | --- |
| Mice/group | N = 7 | | N = 7 | | N = 7 | | N = 7 | |
|  | Mean | SD | Mean | SD | Mean | SD | Mean | SD |
| ALB (g/dL) | 2.8 | 0.8 | 3.2 | 0.4 | 3.3 | 0.1 | 3.9 | 0.7 |
| AST (IU/L) | 254.6 | 31.8 | 210.3 | 17.1 | 228.5 | 10.0 | 215.5 | 6.2 |
| ALT (IU/L) | 46.2 | 5.4 | 48.0 | 2.6 | 52.4 | 4.7 | 42.8 | 2.3 |
| TBIL (mg/dL) | 0.1 | 0.0 | 0.1 | 0.0 | 0.1 | 0.0 | 0.2 | 0.1 |
| BUN (mg/dL) | 24.7 | 3.0 | 21.9 | 1.5 | 22.0 | 3.6 | 23.5 | 1.6 |
| CREAT (mg/dL) | 0.1 | 0.0 | 0.1 | 0.0 | 0.1 | 0.0 | 0.1 | 0.0 |
| PHOS (mg/dL) | 9.6 | 0.5 | 9.7 | 0.5 | 9.5 | 0.4 | 9.2 | 0.6 |
| GLUC (mg/dL) | 96.7 | 4.5 | 100.4 | 3.8 | 95.0 | 3.0 | 98.7 | 3.2 |
| Ca^2+^ (mg/dL) | 8.0 | 0.8 | 10.1 | 1.2 | 9.9 | 0.3 | 9.8 | 0.8 |
| Mg^2+^ (mEq/L) | 1.6 | 0.3 | 1.4 | 0.2 | 1.6 | 0.2 | 1.6 | 0.4 |
| Na^+^ (mEq/L) | 137.6 | 20.9 | 165.3 | 12.6 | 156.3 | 9.5 | 154.3 | 16.6 |
| K^+^ (mEq/L) | 9.5 | 0.7 | 9.3 | 0.6 | 10.6 | 1.0 | 9.1 | 0.5 |
| Cl^-^ (mEq/L) | 93.7 | 12.1 | 123.6 | 10.4 | 105.7 | 8.0 | 125.4 | 14.0 |
| AMYLASE (IU/L) | 767.0 | 37.0 | 786.0 | 60.4 | 765.3 | 24.1 | 724.1 | 32.2 |
| LIPASE (IU/L) | 62.8 | 4.0 | 63.9 | 4.4 | 67.2 | 2.9 | 59.4 | 6.9 |
| CPK (IU/L) | 576.6 | 61.5 | 537.0 | 36.7 | 518.6 | 26.8 | 467.8 | 30.8 |

**TABLE 5. Summary of Day 28 Female Hematology Values**

**Mean + Standard Deviation (SD)**

| Group | 1 - Control | | 2 | | 3 | | 4 | |
| --- | --- | --- | --- | --- | --- | --- | --- | --- |
|  | Mean | SD | Mean | SD | Mean | SD | Mean | SD |
| Mice/group | N = 7 | | N = 7 | | N = 7 | | N = 7 | |
| WBC (10^3^/uL) | 1.2 | 0.2 | 1.2 | .2 | 1.1 | .13 | 1.2 | .11 |
| Neu (/uL) | 1165 | 192 | 1174 | 118 | 1131 | 101 | 1105 | 97 |
| Lymph (/uL) | 304 | 19 | 347 | 44 | 340 | 28 | 334 | 15 |
| Mono (/uL)) | 169 | 34 | 170 | 20 | 138 | 27 | 142 | 23 |
| RBC(10^6^/uL) | 7.8 | .35 | 7.6 | .33 | 7.4 | 0.2 | 8.2 | .6 |
| HGB(g/dL) | 9.8 | .42 | 10.4 | 0.4 | 10.9 | .9 | 11 | 1.4 |
| Platelet Count | 911 | 45 | 928 | 67 | 917 | 66 | 910 | 146 |

**TABLE 6. Summary of Day 28 Male Hematology Values**

**Mean + Standard Deviation (SD)**

| Group | 1 - Control | | 2 | | 3 | | 4 | |
| --- | --- | --- | --- | --- | --- | --- | --- | --- |
|  | Mean | SD | Mean | SD | Mean | SD | Mean | SD |
| Mice/group | N = 7 | | N = 7 | | N = 7 | | N = 7 | |
| WBC (10^3^/uL) | 1.2 | .11 | 1.2 | .16 | 1.1 | .9 | 1.2 | .9 |
| Neu (/uL) | 1174 | 154 | 1142 | 145 | 1149 | 167 | 1083 | 115 |
| Lymph (/uL) | 326.5 | 21 | 331 | 22 | 349 | 25 | 306 | 17 |
| Mono (/uL)) | 134 | 12 | 180 | 15 | 156 | 20 | 149 | 18 |
| RBC(10^6^/uL) | 7.4 | .35 | 7.6 | .4 | 7.8 | .4 | 8 | .6 |
| HGB(g/dL) | 10.2 | .54 | 11 | .6 | 10 | .7 | 9.6 | .4 |
| Platelet Count | 878 | 74 | 936 | 72 | 820 | 59 | 862 | 42 |
